# Exercise barriers self-efficacy: development and validation of a subcale for individuals with cancer-related lymphedema

**DOI:** 10.1186/s12955-015-0223-7

**Published:** 2015-03-18

**Authors:** Jena Buchan, Monika Janda, Robyn Box, Laura Rogers, Sandi Hayes

**Affiliations:** Institute of Health and Biomedical Innovation, School of Public Health and Social Work, Queensland University of Technology, Kelvin Grove, QLD 4059 Australia; Queensland Lymphoedema and Breast Oncology Physiotherapy, Grange, QLD 4051 Australia; Department of Nutrition Sciences, University of Alabama at Birmingham, Birmingham, AL 35233 USA

**Keywords:** Lymphedema, Self-efficacy, Barriers, Cancer, Exercise, Physical activity

## Abstract

**Background:**

No tool exists to measure self-efficacy for overcoming lymphedema-related exercise barriers in individuals with cancer-related lymphedema. However, an existing scale measures confidence to overcome general exercise barriers in cancer survivors. Therefore, the purpose of this study was to develop, validate and assess the reliability of a subscale, to be used in conjunction with the general barriers scale, for determining exercise barriers self-efficacy in individuals facing lymphedema-related exercise barriers.

**Methods:**

A lymphedema-specific exercise barriers self-efficacy subscale was developed and validated using a cohort of 106 cancer survivors with cancer-related lymphedema, from Brisbane, Australia. An initial ten-item lymphedema-specific barrier subscale was developed and tested, with participant feedback and principal components analysis results used to guide development of the final version. Validity and test-retest reliability analyses were conducted on the final subscale.

**Results:**

The final lymphedema-specific subscale contained five items. Principal components analysis revealed these items loaded highly (>0.75) on a separate factor when tested with a well-established nine-item general barriers scale. The final five-item subscale demonstrated good construct and criterion validity, high internal consistency (Cronbach’s alpha = 0.93) and test-retest reliability (ICC = 0.67, p < 0.01).

**Conclusions:**

A valid and reliable lymphedema-specific subscale has been developed to assess exercise barriers self-efficacy in individuals with cancer-related lymphedema. This scale can be used in conjunction with an existing general exercise barriers scale to enhance exercise adherence in this understudied patient group.

## Background

Cancer-related lymphedema is reported as one of the most feared and problematic cancer survivorship concerns [1,2]. A potentially chronic condition, it typically presents as swelling in the limbs, trunk, head, neck or groin, depending on the cancer type. Lymphedema following breast cancer occurs in approximately 20% of women within 18 months of treatment [3], with additional new cases presenting beyond this period [2,4]. While incidence rates for lymphedema in other cancers are limited, a meta-analysis found that, overall, approximately 15% of those with melanoma, sarcoma, genitourinary, gynecological or head/neck cancer subsequently developed cancer-related lymphedema [5].

Participating in regular exercise during and following cancer treatment is considered effective for counteracting treatment-related morbidity, improving function and quality of life, and possibly improving cancer-specific and overall survival [6-9]. Exercise may also help manage lymphedema, but research is predominately limited to breast cancer-related lymphedema [10]. However, despite growing evidence on the importance of engaging in exercise post-cancer, findings from breast cancer studies suggest that approximately 55% do not engage in nationally recommended levels of physical activity [11,12], and nearly 60% report declines in physical activity following their cancer diagnosis [13]. Further, the proportion of women engaging in sufficient levels of physical activity is even lower for women who have lymphedema [14,15]. Yet, participation in physical activity has been associated with less exacerbation of lymphedema-related symptoms in breast cancer survivors [16]. Understanding exercise barriers and self-efficacy for individuals with cancer-related lymphedema will aid international physical activity behaviour change strategies post-cancer diagnosis. Moreover, this information can be used to enhance adherence in the exercise and cancer efficacy trials needed in those cancer types for which less is known about the effects of exercise on lymphedema (e.g., gynaecologic, head and neck).

Exercise barriers self-efficacy is a term used to describe the confidence to overcome barriers and partake in exercise [17], with higher physical activity levels observed in individuals reporting greater self-efficacy to overcome such barriers [18-20]. Common general exercise barriers identified by healthy and clinical populations include time, motivation, social support and weather [17,21-24]. These common barriers may be even greater for cancer survivors due to potentially increased time pressures created by cancer-related medical appointments and treatment requirements [25]. However, cancer survivors also encounter unique barriers to exercise that arise as a consequence of their cancer and its treatment. These may include the presence of treatment-related side effects such as nausea or fatigue, reduced functional capacity or uncertainty about what exercise is safe [15,24,26]. Lymphedema-related barriers, such as the presence of swelling, pain and altered sensation in the affected body area, as well as feelings of fear and uncertainty about making the lymphedema worse, may also exist [15,27,28]. While a scale exists measuring the impact of general barriers on exercise barriers self-efficacy, there is no current tool including lymphedema-related exercise barriers. Therefore, the purpose of this study was to develop, validate and assess the reliability of a lymphedema-specific subscale for measuring exercise barriers self-efficacy in individuals with cancer-related lymphedema.

## Methods

Ethical approval for this study was obtained from the Queensland University of Technology Research Ethics Unit, Brisbane, Australia (Approval # 1100001471). This research has been performed in accordance with the ethical standards laid down in the 1964 Declaration of Helsinki.

### Scale development

A convenience sample of men and women with cancer-related lymphedema was recruited through a local private physiotherapy practice specializing in treatment of lymphedema. Eligibility criteria included a diagnosis of secondary lymphedema due to cancer treatment. Eligible clinic patients were mailed a study information letter and questionnaire by clinic staff to ensure researcher blinding and patient confidentiality. Invited patients were informed that participation was voluntary and could not be tracked, and consent was implied by return of the questionnaire in the provided reply-paid envelope. Participants were also given the opportunity to provide details if they wished to be contacted about providing scale feedback and other future research. A follow-up letter and additional copy of the questionnaire was sent out approximately one month after the initial mailing to maximise response rate.

Initially, ten lymphedema-specific barriers were included. These barriers were identified following review of qualitative and quantitative exercise and lymphedema research [10,27,29-33], and consultation with experts (i.e., allied health professionals and researchers experienced in exercise and lymphedema; backgrounds in exercise science, psychology, lymphedema management, cancer survivorship and physiotherapy). The barrier identification process highlighted that individuals with cancer-related lymphedema face condition-related barriers, as well as general exercise barriers. Therefore, the lymphedema-specific items were designed to be used as a subscale for a previously validated nine-item exercise barriers self-efficacy scale assessing general barriers (internal consistency, Cronbach’s alpha = 0.95; test-retest reliability, *r* = 0.89, p < 0.001) [17], which was also included in the survey. The format of the ten-item lymphedema-specific subscale followed the same format as this pre-existing general self-efficacy validated scale [17]. Therefore, participants were asked to indicate their confidence to overcome barriers (nine general and ten lymphedema-specific) on a scale ranging from 0% (not at all confident) to 100% (extremely confident), with 10% intervals. As is standard procedure for the general self-efficacy scale, responses were then categorised as 0-20% = not at all confident; 20-40% = slightly confident; 40-60% = moderately confident; 60-80% = very confident; 80-100% = extremely confident. Additionally, a follow-up survey mail-out was done to participants that provided contact details expressing interest in future research participation, enabling participants to provide feedback on scale structure and whether there were any relevant barriers missed.

### Scale refinement

Construct validity of the ten-item lymphedema-related subscale was assessed by measuring its correlation with the pre-existing general barriers scale [17]. A principal components analysis was done to determine if all ten items loaded on a single factor. Data from validity testing and factor analysis were then used, in conjunction with participant feedback, to help determine which items to include in the final subscale.

### Testing of final scale

Once revised, validity and reliability testing was completed on the revised lymphedema-specific subscale using a different convenience sample of women with stable, unilateral breast cancer-related lymphedema. These participants were women who had partaken in previous research studies conducted by study investigators, and who had notified us that they were interested in participating in future research. Participants completed the final scale on two occasions, with a two-week interval before repeat assessment. To conduct validity testing, three additional self-report questionnaires were completed, on quality of life, upper-body function and physical activity levels. Quality of life was measured using Functional Assessment of Cancer Therapy-Breast + 4 (FACT-B + 4). This scale, designed specifically for breast cancer patients, has been shown to have sufficient test-retest reliability (r = 0.97) and good internal consistency (alpha coefficient = 0.62 to 0.88) [34]. Upper-body function was assessed using the Disabilities of the Arm, Shoulder and Hand (DASH) questionnaire, a validated tool that measures the impact of upper-limb limitations on daily life (alpha = 0.96) [35]. The Active Australia survey was used to assess weekly physical activity participation. Total number of sessions and time spent engaged in activity is calculated and reported as “sedentary” (physical activity = 0 minutes), “insufficient” (physical activity < 150 min OR physical activity ≥ 150 min and number of sessions < 5) or “sufficient” (physical activity ≥ 150 min AND number of sessions ≥ 5). The Active Australia survey has demonstrated good test-retest reliability and validity (intra-class correlations = 0.71-0.86; Spearman’s Rho = 0.54-0.77; Kappa statistics = 0.52) [36,37].

### Statistical analysis

Frequencies were run for all items on the original and final version of the lymphedema-specific subscale, to check for any invalid values and potential outliers. As is standard procedure for the general exercise barriers self-efficacy scale [38], if participants missed individual items, and as long as responses for less than 33% of items were missing, the mean of remaining items was imputed to allow calculation of the total scale score. When participants missed more than 33% of items, their data were excluded from analysis.

Principal components analysis with direct oblimin rotation was completed for final subscale analyses. To validate the subscale, we correlated scores from the lymphedema-specific subscale with the ten-item general barriers scale. Correlations were also calculated between the lymphedema-specific subscale and quality of life (FACT-B + 4;) and upper-body function (DASH) (construct [discriminant] validity) scores, as well as physical activity levels (criterion validity; one-way ANOVA). We expected low correlations between the scale and quality of life and upper-body function, given the differences in these constructs. However, higher self-efficacy levels were expected in participants reporting higher physical activity levels. The internal consistency (i.e., Cronbach’s coefficient alpha) and test-retest reliability statistics (i.e., intraclass correlation coefficients [ICC] and paired-sample t-tests) were also calculated. Intraclass correlation coefficients were used to determine how well repeated measurements resembled one another (i.e., how consistent participants were in responding), and paired-sample t-tests were used to examine if changes in response were statistically significant from initial to repeat assessment for any item.

## Results

### Participant characteristics

**Figure 1:**
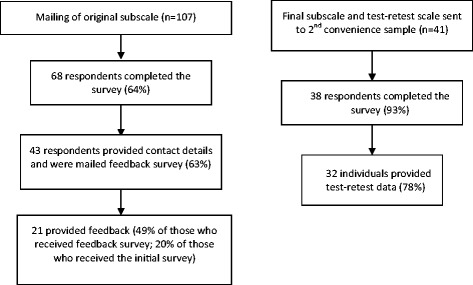
**Participant response flow.**

Responses from 68 (64%) of the original sample were received, with their data being used for psychometric assessment of the original lymphedema-specific subscale (ten items), while 38 participants (93% of the second sample) provided data for psychometric testing (five items) of the revised subscale. The data and feedback collection processes are outlined in Figure 1.

Table 1 presents an overview of participant demographic and medical characteristics of the two convenience samples. Briefly, sample one and two were similar in age (mean [95%CI]: 59.6 [57.1,62.1] and 56.3 [53.1,59.5] years, respectively) and the majority were employed (64.7% and 57.9%, respectively) and lived with a partner (married, de facto or serious partner; 77.9% and 63.2%, respectively). The majority of respondents reported participating in some weekly physical activity (91.2% and 86.8%, respectively). Key differences between samples were that all individuals from the second sample had lymphedema following breast cancer, compared with only 66% of those in the first sample. Further, compared with sample two, more respondents in sample one had lymphedema for longer than five years (sample one: 25%; sample two: 13%).

**Table 1:** **Descriptive characteristics of participants**

**Variable**	**Original scale sample**	**Final scale sample**
	**(N = 68)**	**(N = 38)**
	**n (%)**	**n (%)**
Age (year)		
Age, mean (95% CI)	59.6 (57.1,62.1)	56.3 (53.1,59.5)
Gender		
Male	5 (7.4)	0 (0)
Female	63 (92.6)	38 (100)
Marital Status		
Married/de facto	53 (77.9)	24 (63.2)
Single/widowed/divorced	15 (22.1)	14 (36.8)
Employment status		
Paid employment	44 (64.7)	22 (57.9)
Unemployed/retired/unpaid work	24 (35.3)	16 (42.1)
Total physical activity^a^		
Sedentary	6 (8.8)	5 (13.2)
Insufficient	21 (30.9)	13 (34.2)
Sufficient	41 (60.3)	20 (52.6)
Cancer		
Breast	45 (66.2)	38 (100)
Gynaecological	14 (20.6)	0 (0)
Other (skin, bowel, prostate)	9 (13.2)	0 (0)
Duration with lymphedema		
<2 years	26 (38.2)	16 (42.1)
2-5 years	25 (36.8)	17 (44.7)
>5 years	17 (25.0)	5 (13.2)

CI = confidence intervals; min = minutes.

^a^sedentary = no weekly physical activity; insufficient = < 150 min OR ≥ 150 min and < 5 sessions per week; sufficient = ≥ 150 min and ≥ 5 sessions per week.

### Original subscale

Results from the principal components analysis on the original ten-item lymphedema-specific subscale (Table 2) suggested items loaded on two factors, which accounted for 73.5% of the total variance. Eight items loaded well on the first factor (0.67 to 0.88), and two items (“when I am lifting/moving heavy objects a few times” and “when I am lifting/moving light objects repetitively”) loaded on the second factor (0.90 and 0.95). Scores on the general and lymphedema-specific scales were strongly correlated, indicating good construct validity (Pearson’s *r* = 0.72, p < 0.01).

**Table 2:** **Original lymphedema-specific exercise barriers self-efficacy subscale**

**Using the scale from 0-100%, indicate how confident you are that you could exercise in each of the following situations. Even if you are not currently exercising, please read and respond to each question by circling one number for each situation.**											
	**Not at all confident**		**Slightly confident**		**Moderately confident**			**Very confident**		**Extremely confident**	
When I am worried about my appearance.	0%	10%	20%	30%	40%	50%	60%	70%	80%	90%	100%
When my affected body segment feels heavy.	0%	10%	20%	30%	40%	50%	60%	70%	80%	90%	100%
When my affected body segment is painful.	0%	10%	20%	30%	40%	50%	60%	70%	80%	90%	100%
When my affected body segment is numb or tingling.	0%	10%	20%	30%	40%	50%	60%	70%	80%	90%	100%
When I fear making my lymphoedema worse.	0%	10%	20%	30%	40%	50%	60%	70%	80%	90%	100%
When I am unsure what exercise advice to follow.	0%	10%	20%	30%	40%	50%	60%	70%	80%	90%	100%
When I am not certain if I am doing an exercise correctly.	0%	10%	20%	30%	40%	50%	60%	70%	80%	90%	100%
When I am lifting/moving heavy objects a few times.	0%	10%	20%	30%	40%	50%	60%	70%	80%	90%	100%
When I am lifting/moving light objects repetitively.	0%	10%	20%	30%	40%	50%	60%	70%	80%	90%	100%

Twenty-one respondents (20%) from sample one provided feedback on the subscale and suggested inclusion of items that dealt with lack of time and/or motivation, work and family commitments, exacerbation of lymphedema symptoms, embarrassment, pain, lack of confidence, and limited advice following treatment. Since most of these suggestions are already covered by the general self-efficacy scale, it highlighted the necessity for concurrent assessment of general and lymphedema-specific exercise barriers self-efficacy. Respondents also suggested clarifying the question on embarrassment about appearance (whether this was lymphedema-related or general, due to for example obesity or being unfit) and suggested defining the term ‘exercise’, which is used in the questionnaire instructions. Of note, 4 of the 21 respondents providing feedback had a cancer type other than breast (i.e., gynecological, bowel) with suggestions for additional barriers being similar for breast and non-breast cancer survivors.

### Final subscale

By considering results from the factor analysis and participant feedback, the original ten items in the lymphedema-specific subscale were reduced to a five-item subscale (Table 3) to be used in conjunction with the nine-item general exercise barriers self-efficacy scale. Three of the five items reflect original, unchanged items: “when I fear making my lymphedema worse”, “when I am not certain I am doing an exercise correctly”, and “when I am unsure what exercise advice to follow”. In line with participant feedback, the appearance barrier question was reworded to clarify that it was related to worry regarding swelling and compression garment use (“when I am worried about my appearance [e.g., due to swelling and/or compression garment]”) and the four original items about side-effects as a barrier (heaviness, swelling, numbness/tingling and pain) were collapsed into a single item. The two items loading on a separate factor in the original subscale principal components analysis were removed, also guided by participant feedback suggesting these items were only relevant in certain situations (i.e., during resistance-based exercise).

**Table 3:** **Final lymphedema-specific exercise barriers self-efficacy subscale**

**Using the scale from 0-100%, indicate how confident you are that you could exercise in each of the following situations (‘exercise’ is planned physical activity undertaken for health benefits, e.g. lifting weights, planned walks, swimming). Even if not currently exercising, please read and respond to each question by circling one number for each situation.**											
	**Not at all confident**		**Slightly confident**		**Moderately confident**			**Very confident**		**Extremely confident**	
When I am worried about my appearance (e.g. due to swelling and/or compression garment).	0%	10%	20%	30%	40%	50%	60%	70%	80%	90%	100%
When I am experiencing lymphoedema-related symptoms (e.g. pain, heaviness, numbness/ tingling, swelling).	0%	10%	20%	30%	40%	50%	60%	70%	80%	90%	100%
When I fear making my lymphoedema worse.	0%	10%	20%	30%	40%	50%	60%	70%	80%	90%	100%
When I am unsure what exercise advice to follow.	0%	10%	20%	30%	40%	50%	60%	70%	80%	90%	100%
When I am not certain if I am doing an exercise correctly.	0%	10%	20%	30%	40%	50%	60%	70%	80%	90%	100%

Scale structure: Results of the principal components analysis applied to data collected from the second sample showed that items loaded on two significant factors (that is, the nine-item general exercise barriers self efficacy items loaded on factor one and the five items from the lymphedema-specific subscale loaded on factor two), and together accounted for 76.6% of the total variance. Factor loadings for the nine items of the general self-efficacy scale [30] and the five-item lymphedema-specific subscale ranged from 0.60 to 0.95 and 0.64 to 0.97, respectively.

Validity: The final lymphedema subscale was strongly correlated with the ten-item general barriers scale, indicating good construct validity (Pearson’s *r* = 0.61, p < 0.01). The lymphedema-specific scale was poorly associated with quality of life (FACT-B + 4) and upper-body function (DASH) (Pearson’s *r* = 0.31 and Pearson’s *r* = −0.34, respectively). Criterion validity testing showed individuals classified as both insufficiently active (>0 min but < 150 min OR ≥ 150 min and < 5 sessions weekly) and sufficiently active (≥150 min and ≥ 5 sessions weekly) had higher self-efficacy scores (mean [SD]: 62.1 [15.7] and 56.7 [23.9], respectively) than individuals performing no physical activity (42.4 [30.4]), though this difference was not supported statistically (p = 0.24).

Reliability: Cronbach’s alpha score of the final subscale was high (alpha = 0.93), indicating strong internal consistency. Participants answered the scale consistently at the test and re-test time-points, with their overall score of the first and second completion correlating highly (ICC = 0.67, p < 0.001). Test-retest correlations of individual items ranged from 0.44 to 0.65 (p < 0.01 for all). Paired-sample *t*-test showed no statistically significant change from initial to repeat assessment for any item (Table 4).

**Table 4:** **Paired-sample** ***t*** **-test values for test-retest reliability of final five-item lymphedema-specific subscale**

**Scale item**	**Test-retest mean difference (95% CI)**	**t-score**	***p*** **-value**
Total scale	0.79 (−5.28, 6.87)	−0.266	0.792
When I am worried about my appearance	−3.75 (−10.53, 3.03)	1.129	0.268
When I am experiencing lymphedema-related symptoms	3.75 (−5.49, 12.99)	−0.828	0.414
When I fear making my lymphedema worse	2.50 (−6.84, 11.84)	−0.546	0.589
When I am unsure what exercise advise to follow	2.09 (−5.15, 9.33)	−0.589	0.560
When I am not certain if I am doing an exercise correctly	−0.63 (−8.50, 7.25)	0.162	0.872

CI = confidence intervals.

## Conclusions

This study has led to the development of a valid and reliable exercise barriers self efficacy scale to assess confidence in ability to exercise when faced with barriers experienced by individuals with cancer-related lymphedema. This scale combines ten general exercise barriers, a pre-existing scale developed by Rogers and colleagues [17], with five lymphedema-specific barriers, a subscale developed in this study. The subscale correlated highly with the general self-efficacy scale, but formed a distinct separate factor, indicating the importance of lymphedema-specific barriers to exercise.

This study addresses a key gap in the evidence, as previously only scales that assess general exercise barriers in breast cancer survivors [17,39] or ‘healthy’ populations [40] were available. Initial validity testing conducted as part of this study is promising. As has been demonstrated in general [18] and cancer [41] populations with the general exercise barriers self-efficacy scale, participants who had lower self-efficacy engage in less activity compared with those who report higher self-efficacy. Importantly, the scale does not overlap with the measurement of other constructs, such as quality of life and upper-body function.

Reliability testing yielded an alpha coefficient of 0.93 for the final lymphedema-specific subscale. This is a high value and similar to those found for other exercise barriers self-efficacy scales [17,39]. It may indicate that the five items within the lymphedema-specific scale are too similar and further items are needed that cover other lymphedema-related barriers not yet described, or may be a reflection of the homogeneity of samples in the previous, as well as our, studies. Nevertheless, additional barriers were not identified when feedback was obtained from the sample completing the original ten-item subscale. Test-retest reliability for the subscale was good (ICC = 0.67), with values for individual items ranging between 0.44 and 0.65. The overall mean differences for each item from test to retest ranged from 0.6 to 3.8 points and all responses remained within the same category; for example participants were still ‘moderately confident’ on the initial and re-test scale for any given item. These findings are consistent with those found by Rogers and colleagues [17], in their validity and reliability testing of the original nine-item general barriers scale. In practice, these findings suggest that the total scale score is robust, but that it is not ideal to focus on results from any one specific item within the scale.

It should be noted that scale construction and validity and reliability testing was undertaken using two, relatively small (N = 68 and N = 38), convenient and likely homogenous samples (66% and 100% of sample one and two respectively were women with breast cancer-related lymphedema and >75% of the samples had lymphedema for less than five years). Additionally, no data were available on non-respondents, so it cannot be determined how representative this sample was of the general lymphedema population. Both sample size and homogeneity have important implications for results of the principal components analysis, with further testing warranted in a larger, more diverse population to confirm items still load on a single lymphedema barrier self-efficacy factor. Nonetheless, the initial subscale development process collected barriers reported by survivors with breast and non-breast cancer-related lymphedema. Specifically, we were able to make use of qualitative data collected from focus groups and telephone interviews exploring how individuals with cancer-related lymphedema (16 in focus groups of 2–4 participants, 13 completing telephone interviews) construct their experience in daily life [33]. Participants in this qualitative work included men and women with lymphedema following treatment for breast, gynecological or ‘other’ cancers. As part of this qualitative work, participants were questioned about potential barriers of participation in physical activity with issues raised incorporated into the original subscale. This information was further supplemented by consultation of specialists in the field (dealing with patients with upper- and lower-limb lymphedema), an extensive literature search and incorporation of written participant feedback following completion of our original subscale. Our results showed, in looking at characteristics of participants providing feedback, there were no key differences in feedback received from people with breast versus other cancer-related lymphedema. As such, it is feasible that the lymphedema-specific barriers included are relevant to, and representative of the barriers faced by, individuals with lymphedema following cancer other than breast.

Using the new lymphedema-specific subscale along with the existing general barriers self-efficacy scale by Rogers and colleagues’ [17] will allow healthcare professionals and patients to identify low self-efficacy for overcoming exercise barriers when cancer-related lymphedema is a concern. In turn, this should assist patients and their support team in identifying ways to overcome barriers and improve exercise uptake and adherence. The addition of the lymphedema-specific scale in future exercise interventions involving people with cancer-related lymphedema may also be useful to help guide individual program design. Baseline measurements of overall self-efficacy levels at the start of an exercise intervention can be used to identify participants that may be at risk of poorer adherence [12,19] and to guide and individualize the level of support during exercise interventions or programing. Alternatively, researchers could assess whether baseline self-efficacy levels influence the effect of exercise interventions or whether participation in an exercise intervention has the potential to increase self-efficacy levels. In summary, this work extends the research in exercise barriers self-efficacy in the general cancer population into the understudied area of cancer-related lymphedema. In doing so, future research in this area could assist those with cancer-related lymphedema to become more confident in overcoming barriers and engaging in exercise, ultimately improving their physical and psychosocial well-being.
